# Optimizing procedures for ocular radiation injury studies in the tree shrew

**DOI:** 10.37349/etat.2025.1002352

**Published:** 2025-12-01

**Authors:** Lauren A. Dalvin, Kjersten J. Anderson, Tommy A. Rinkoski, David R. Miley, Hien Ong, Angela M. Schechinger, Cassandra A. Fjeld, Catherine R. Leblond, Mackenzie K. Keown, Sierra D. Palmer, Danielle M. Burgenske, Brett L. Carlson, Lauren L. Ott, Brian C. Samuels, Michael F. Romero, Jann N. Sarkaria, Felicia Duke Boynton, Gavin W. Roddy

**Affiliations:** IRCCS Istituto Romagnolo per lo Studio dei Tumori (IRST) “Dino Amadori”, Italy; ^1^Departments of Ophthalmology, Mayo Clinic, Rochester, MN 55905, USA; ^2^Departments of Medical Oncology, Mayo Clinic, Rochester, MN 55905, USA; ^3^Departments of Comparative Medicine, Mayo Clinic, Rochester, MN 55905, USA; ^4^Departments of Surgery, University of Minnesota, St. Paul, MN 55108, USA; ^5^Departments of Veterinary Population Medicine, University of Minnesota, St. Paul, MN 55108, USA; ^6^Departments of Radiation Oncology, Mayo Clinic, Rochester, MN 55905, USA; ^7^Department of Ophthalmology and Visual Sciences, University of Alabama at Birmingham, Birmingham, AL 35294, USA; ^8^Departments of Physiology and Biomedical Engineering, Mayo Clinic, Rochester, MN 55905, USA

**Keywords:** tree shrew, radiation, ocular radiation injury, radiation retinopathy, radiation optic neuropathy

## Abstract

Radiation exposure to the eye during cancer treatment can lead to ocular radiation injury (ORI), a devastating condition that can have a profound and permanent impact on vision-related quality of life. Rodent models do not have adequate ocular anatomy to accurately simulate human ORI, and modeling in non-human primates is limited by logistical and ethical concerns. To improve future translational research investigating ways to treat or prevent ORI, we developed protocols for a tree shrew model of ORI. Northern tree shrews (*Tupaia belangeri*) were obtained by our laboratory. Custom housing and handling methods were developed, including custom body suits to maintain the tree shrew’s body temperature during procedures. Radiation delivery was optimized to accurately deliver radiation, and imaging was performed to observe fundus changes from ORI. Optimization of tree shrew handling, housing, anesthesia approaches, radiation delivery, and clinically-relevant ocular imaging permitted successful induction and assessment of ORI in tree shrews. With these protocols, tree shrews can be used as a highly relevant model organism with key anatomic features similar to humans to study ORI.

## Introduction

Radiation exposure can have a variety of adverse effects on the eye, and some of the most devastating side effects cause permanent damage to retinal and optic nerve tissues, resulting in vision loss. Within 5 years following exposure, direct radiation to the eye can cause ocular radiation injury (ORI), leading to visual acuity of 20/200 or worse, which meets the criteria for legal blindness in the affected eye(s) [1, 2]. High levels of radiation exposure are most often encountered in the setting of cancer treatment. By 2030, the number of radiation-treated cancer survivors is expected to reach 4.17 million [3], and given the direct impact of ORI on daily quality of life [4], research is needed to develop strategies for the prevention and treatment of ORI.

A major barrier to studying ORI in the laboratory has been a lack of representative animal models. Most small animal studies for ORI have utilized the rat, but the model remains poorly characterized with respect to clinically relevant retinal imaging and mechanisms of disease induction [5]. Furthermore, and more importantly, the anatomy of the rat does not permit adequate replication of human eye disease. Rats have a rod-dominant retina [6], lack a lamina cribrosa [7], and permit clinical imaging at only 10% of the quality seen when imaging humans [8].

Northern tree shrews (*Tupaia belangeri*) are one of the closest living relatives to primates [9] and have emerged as a small-in-size model with primate-like ocular anatomy to study a variety of eye diseases [9–12]. Compared to rodents, tree shrews have several advantages as a model for ocular pathology. Their cone-dominant retina with superior visual acuity and near-emmetropic refractive error [13] provides a better avenue to study macular pathology [10], and the presence of a load-bearing lamina cribrosa is required for the study of multiple optic neuropathies [14]. Improved imaging optics of the tree shrew eye also permit high-resolution in vivo retinal imaging [10]. Despite these advantages, the expected expansion of tree shrew research has been attenuated as the tree shrew requires unique environmental and handling approaches. To help establish a novel model for improving translation of ORI studies that will facilitate future quality of life sustaining research for cancer patients, we herein report our experience developing protocols for handing and housing as well as inducing and monitoring ORI in the tree shrew. These models will support translational research, including the development and validation of novel therapeutic strategies for improving vision preservation in patients undergoing radiation therapy.

## Materials

### Housing and husbandry

**Figure 1:**
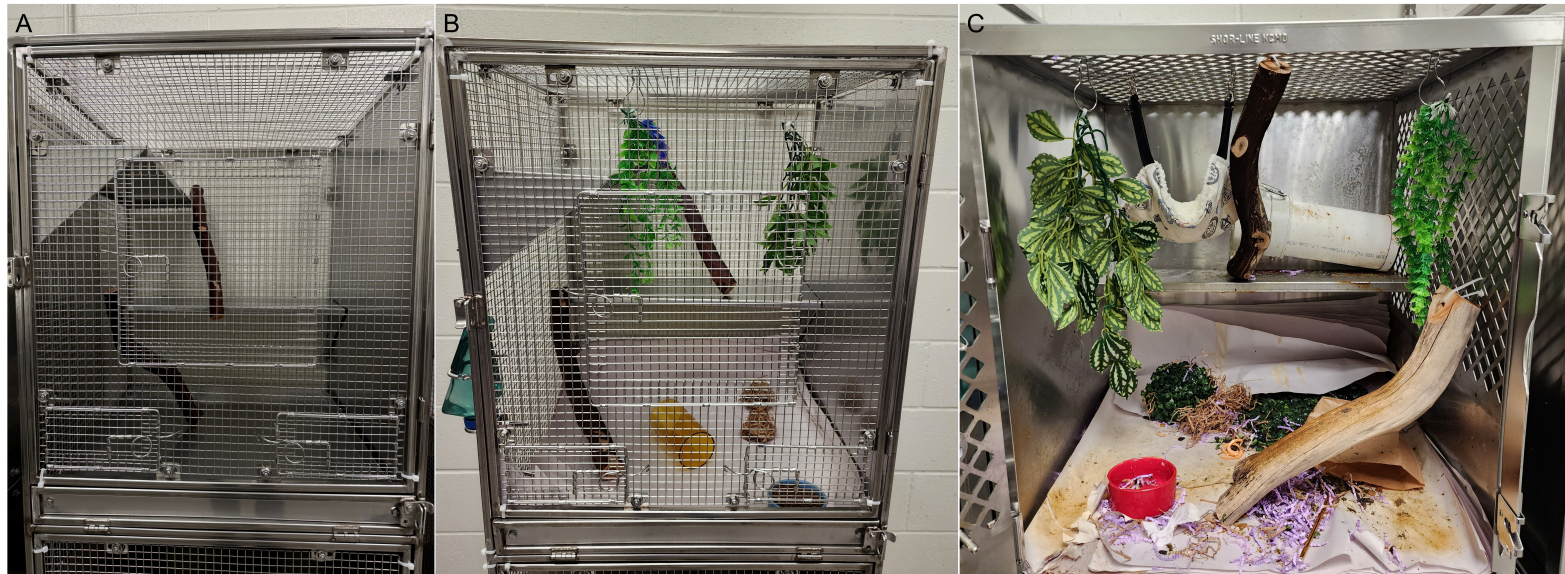
**Tree shrew housing and environmental enrichment.** (**A**) Customized tree shrew housing included 29 inches of vertical space, two resting boards, and mesh side panels for climbing. (**B**) Tree shrews were provided with a variety of toys for environmental enrichment, including balls, aquarium plants, hanging jingle toys, Manzanita sticks, loosened Bed-r’Nest pucks, and a PVC nesting tube. (**C**) An enrichment environment can be seen following use.

Custom stainless steel tree shrew cages were purchased from the Tree Shrew Cages Collection (Safe Haven Lab Cages) with some modifications (Figure 1A). Cage size was 24 × 24 × 29 inches, providing 29 inches of vertical space and 576 square inches of floor space. Cages were designed to incorporate sufficient light exposure. Two raised resting boards were present, and there was one solid side wall for reduced visual contact with neighboring animals, and one mesh side panel for climbing. Not pictured was a grid floor in each cage. Doors included animal and food bowl access. Tree shrews were fed 2072 Teklad Global Ferret Diet (Inotiv) ad lib in stainless steel bowls or ceramic crocks, with municipal or reverse osmosis water ad lib in plastic bottles with sipper tubes mounted outside of each cage. Daily enrichment foods included fresh vegetables, peanuts, sunflower seeds, hardboiled egg, Greek yogurt, and dried mealworms. Occasional treats included fresh or canned fruits, canned mealworms, canned or dried crickets, and fruit juice given via syringe.

**Figure 2:**
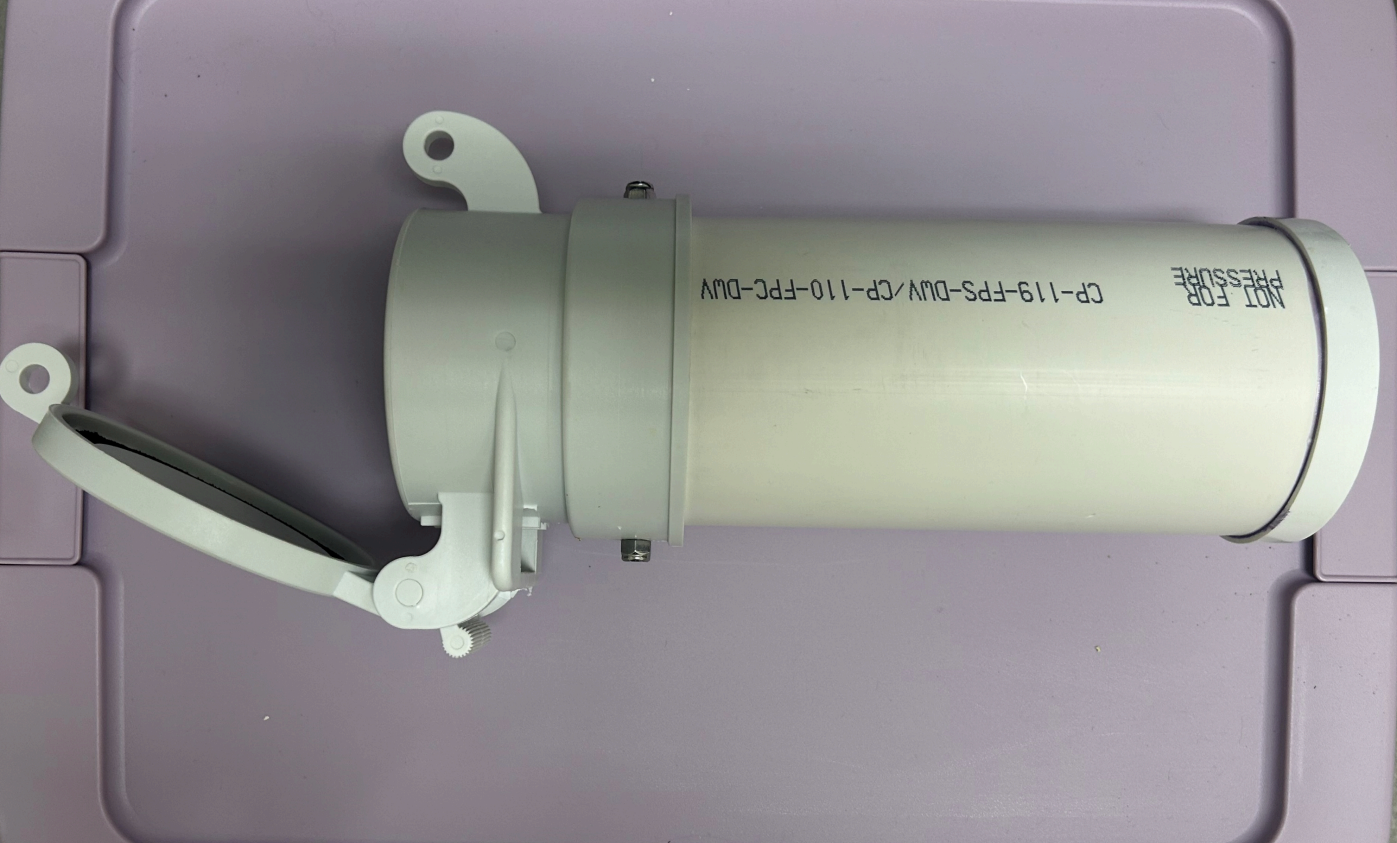
**Tree shrew nesting.** A PVC nest tube was provided for tree shrew sleeping quarters and handling of conscious animals.

Bedding and nesting material were provided in each cage, including a PVC nest tube of 11 or 13 inches in length with a 4-inch inner diameter and a solid cap on one end (FootLoose RV Site Sewer Cap, 4” White Female, Enviro Design Products) (Figure 2). Newsprint paper (ULINE) was used to line the wire grid flood and drop pans, and two loosened Bed-r’Nest pucks (The Anderson’s) were included in each cage.

Enrichment was provided (Figure 1B and C). A sound machine (Homedics SoundSleep White Noise Sound Machine, Silver, Small Travel Sound Machine with 6 Relaxing Nature Sounds, Portable Sound Therapy for Home, Office, Nursery, Auto-Off Timer, Homedics) played naturalistic soundscapes, which rotated daily during lights on. Spray paper enrichment items with essential oils were offered weekly (Nature’s Truth Aromatherapy Lavender Essential Oil). Dried herbs were included in enrichment devices (oregano and rosemary, McCormick). Structurally, each cage contained up to two manzanita sticks (18 inches) mounted with zip ties for climbing, perching, and scent marking (Manzanita Wood Gnawing Sticks, Bio-Serv) and up to two aquarium plants for hiding and climbing (PENN-PLAX Aqua-Plants Plastic Aquarium 3 Piece Fish Plant, Green, 16-in height, Chewy.com; KOMODO Climbing Bamboo Aquarium Plant, Medium, Chewy.com; KOMODO Climbing Two Tone Aquarium Plant, Medium-Chewy.com). Rotating toys included balls (Nobbly Wobbly, Large, Assorted Colors, Bio-Serv; Hol-EE Mol-EE, Assorted Colors, Certified, Bio-Serv; Maritown Topiary Ball 2 Pack Artificial Boxwood Balls), plastic round fake plants (Faux Greenery Topiaries for Home Patio Front Porch Garden Decor Indoor Outdoor (2, 5 inches), Amazon.com); a rat tube (Rat Tunnels, Certified, Bio-Serv); destructible containers (e.g., empty glove boxes, pipette tip boxes, paper bags or cups, etc.); destructible substrate (shredded paper, Sani-Chips, Enviro-dri, etc.); and fodder (meal worms, paper straws cut to various lengths). Social enrichment was provided with positive reinforcement, target training using a laser pointer or finger.

### Anesthetic medications and monitoring

Medications for anesthesia induction and maintenance included xylazine [4–6 mg/kg (7.7 mg/mL) intramuscularly in quadriceps or subcutaneously in scruff or flank], midazolam (1–2 mg/kg intramuscularly in quadriceps or subcutaneously in quadriceps, combined with xylazine), maropitant citrate [1 mg/kg (1 mg/mL) subcutaneously in scruff or flank, to prevent vomiting], flumazenil (0.25 mg/kg, as a reversal agent if needed), atipamezole [0.5 mg/kg (0.5 mg/mL), as a reversal agent if needed], and isoflurane (0–5% inhalation via induction chamber or nose cone on non-rebreathing circuit). Intramuscular injections were limited to 0.25 mL per injection site.

Equipment was utilized for monitoring the vital signs of animals under anesthesia. Temperature was monitored via rectal thermometer (Vetcorder, Pro-MAI Animal Health), SpO_2_ via Vetcorder (Pro-MAI Animal Health) or 2500A VET handheld pulse oximeter (Nonin), and heart rate via Vetcorder (Pro-MAI Animal Health) or 2500A VET handheld pulse oximeter (Nonin).

**Figure 3:**
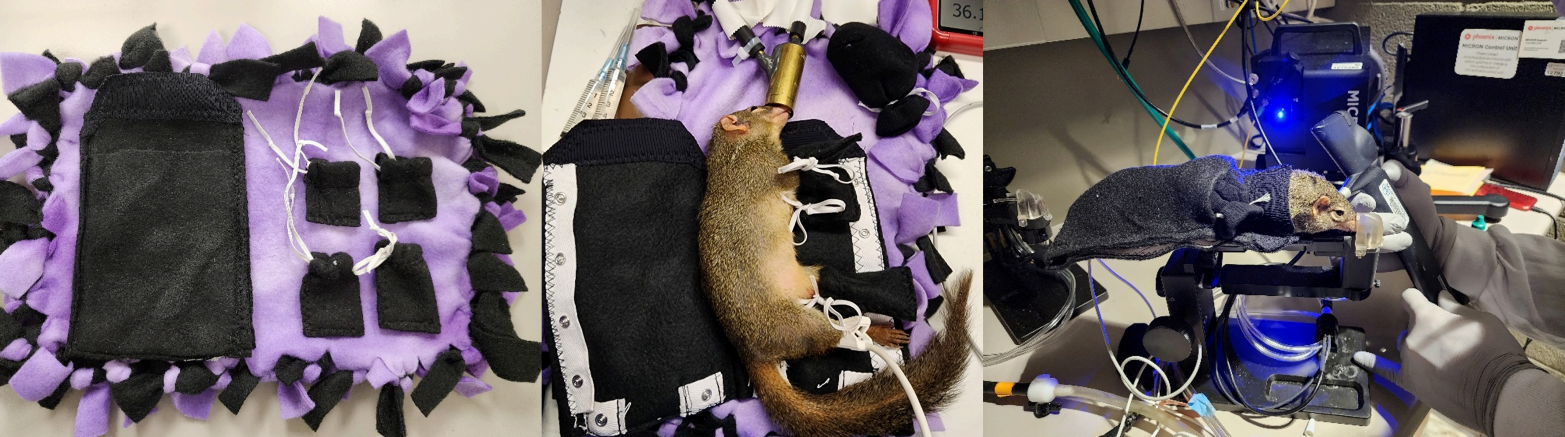
**Custom tree shrew body suits.** Custom body suits with mittens and snap closures were used to prevent heat loss during procedures while maintaining access for monitoring equipment. In the far-right panel, an animal can be seen in a fully closed body suit on the ocular imaging stage with a nose cone for isoflurane delivery. The custom body suit with a narrow head opening allowed easy access for intraocular pressure (IOP) measurements (shown) and multimodal ocular imaging.

To support procedures, custom body suits were made of fleece fabric folded in half to create a rectangular sleeve similar to a sleeping bag (Figure 3). Body suits include a top thinner layer, a middle fleece layer, and a pocket on the outside. The folded edge runs along one side of the animal, and the 3 open sides can be fastened to improve insulation. The top open side for the head is slightly narrowed, and the final design utilized metal snap buttons. Both small and large sizes were created, each with a 0.25-inch thickness and 6-inch length. The small had a 4.5-inch opening at the bottom and a narrower 3.5-inch opening at the top for the head, while the large had a 5.5-inch width at the bottom and a standard 3.5-inch opening at the top. Mittens were made from the same fleece fabric, measuring 2 × 2 inches with an elastic purse string closure.

### IOP assessment

A TonoLAB (iCare, Colonial Medical Supply, Londonderry, NH) on rat setting was used for IOP assessments. Refresh Tears Lubricant Eye Drops (Allergan, Irvine, CA) were used prior to IOP measurement.

### Ocular imaging

Tropicamide (Somerset Therapeutics, LLC, Hollywood, FL) ophthalmic drops were required for dilation of tree shrew eyes prior to imaging, and GenTeal (Alcon Laboratories, Inc., Fort Worth, TX) topical ophthalmic gel was used as a coupling agent during the imaging procedure. The Phoenix MICRON 5 (Phoenix-Micron, Inc., Bend, OR) was used for imaging, including fundus photography, optical coherence tomography (OCT), and fluorescein angiography (FA). For FA, 200 µL of fluorescein dye (1:5 dilution of 100 mg/mL stock) was injected intraperitoneally.

### Radiation delivery

**Figure 4:**
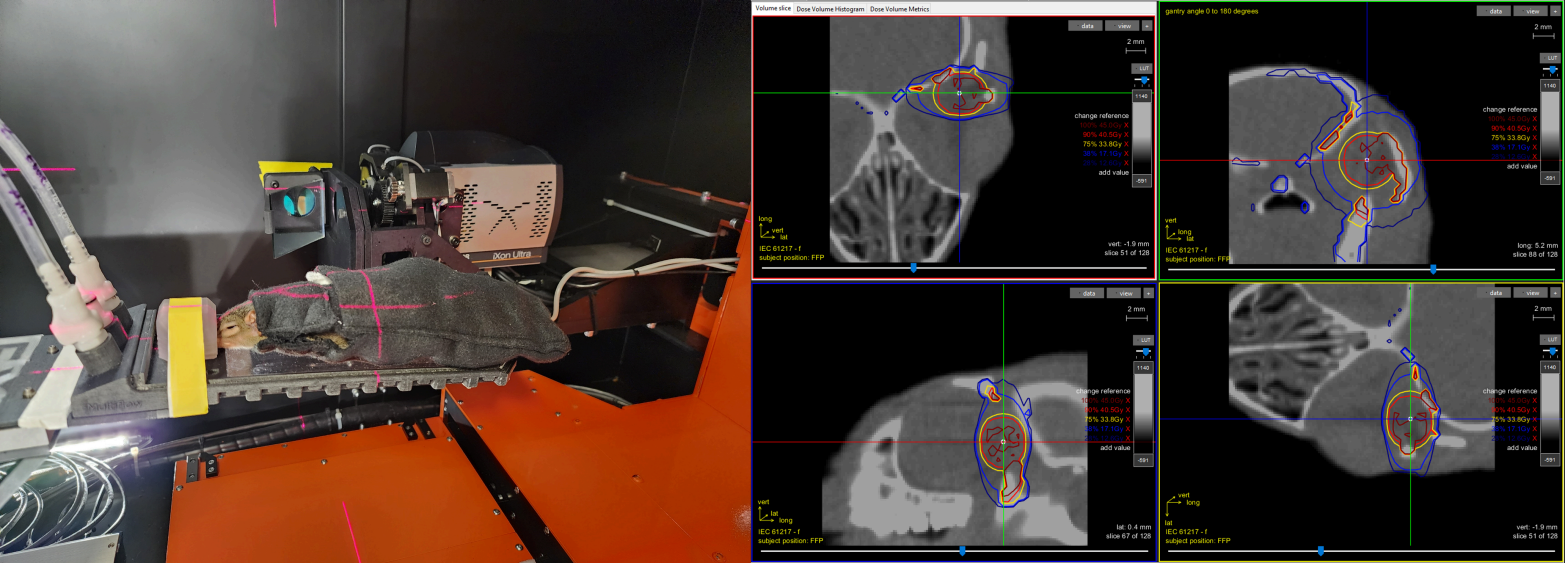
**Radiation setup and targeting.** Tree shrews were mounted on the stage for radiation procedures (left). Radiation targeting was planned using bony landmarks visualized with real-time CT scans (right), with confirmation by imaging the optic nerve and posterior edge of the globe. The software provides a 4-panel view to assist with radiation targeting.

An X RAD SmART irradiator (Precision X-ray Inc., Madison, CT) was used for ocular radiation delivery (Figure 4).

### Intravitreal injection

The impact of drugs can be tested using intravitreal drug delivery to simulate intravitreal injections given to human patients. Lidocaine 4% ophthalmic drops were used for local anesthesia, with 5% povidone iodine for antisepsis. A 0.12 forceps was used to rotate the eye, with a caliper to measure the appropriate injection distance from the limbus and a 33 g needle (TSK Laboratory, Japan) on a 50 µL Hamilton syringe (Hamilton Co., Reno, NV) used to deliver drugs in a volume of 5 µL.

### Euthanasia

For euthanasia, induction was done with 5% isoflurane dosed as for other procedures, followed by intraperitoneal administration of 2 mL euthanasia solution (390 mg/mL pentobarbital). Bilateral pneumothorax was performed after death as a secondary method of euthanasia.

## Procedure

All experiments complied with the Guide for the Care and Use of Laboratory Animals and were approved by the Mayo Clinic IACUC.

### General handling and training

A behavioral management program was implemented to improve the quality of life and to help provide additional opportunities for training and trust building, as the species is known to be particularly sensitive to stressors in captivity. The program included a standardized positive reinforcement training regimen using target training, which was adapted from previously reported non-human primate training protocols [15]. Training sessions were conducted 1 to 3 times per week with each of 12 singly housed tree shrews (6 male, 6 female). Sessions assessed the tree shrews’ approach to handler, affective state, time to task completion, food preferences, and the presence of any stereotypic behaviors or vocalizations. Before starting each training session, the trainer kept a treat (typically broken up mealworms) in hand with the laser pointer in the other and approached the cage to determine if the shrew was willing to participate in a training session. If the shrew approached the front of the cage to accept a treat from the trainer’s hand, training proceeded with the laser pointer session. The laser pointer first targeted a spot on the wall, and the goal was to have the shrew approach the laser and touch it. If the tree shrew approached the target, a vocal bridge (“good job”) marked the behavior, and a reinforcing food item was given. The last step of laser training was having the shrew follow the laser to their upper shelf and into their nesting tube, which was rewarded with a reinforcing food item. Voluntary entry to the nesting tube facilitated low-stress, no-contact handling for husbandry and experimental procedures.

### Housing and husbandry

Tree shrews were housed singly, with visual, auditory, and olfactory contact with other tree shrews housed in the same room. The temperature was set at 75–76°F, with a goal of 30–70% humidity. Tree shrews were maintained on a 12/12-hour light/dark cycle, with lights on at 6 am and off at 6 pm. Paper lining, Bed-r’Nest, water bottles, and food bowls were changed weekly, with weekly sanitization of rotating enrichment toys. Rack, cage, and nesting tube sanitation was done monthly, with separation of cage and tube sanitization by at least one week to maintain animal scent in the home cage. Given the highly conscientious nature of the tree shrews, we recommend limiting handling staff to only a few consistent individuals. We found that consistent, female staff reduced animal distress.

### Anesthesia

Animals were fasted for up to 5 hours prior to procedures. Induction was done using isoflurane followed by a one-time administration of xylazine, maropitant citrate to prevent vomiting, and midazolam. We found that subcutaneous injection of medications was less aversive and had minimal impact on induction times. Warmed subcutaneous fluids were also administered (5 mL Plasmalyte). Isoflurane was delivered continuously throughout any procedures for maintenance of anesthesia. If transport was required to procedural areas, animals were transported in a warmed nest tube inside a small animal carrier. While under anesthesia, temperature, SpO_2_, and heart rate were monitored every 5–15 minutes.

Tree shrews were placed in custom body suits during procedures. Mittens were applied to the feet of anesthetized animals before placing the animal in the body suit. The tail was tucked into the body suit before closure to minimize heat loss. Wires from monitoring devices (e.g., pulse oximeter, thermometer, etc.) can exit the body suit through the spaces between snap buttons on the body suit, and snaps can be opened to access the animal for visual inspection, injections, adjustment of monitoring equipment, and other procedural needs.

### IOP assessment

IOP was assessed shortly following induction of anesthesia using rebound tonometry. After applying topical artificial tears, a TonoLAB rebound tonometer was positioned perpendicularly, approximately 2 mm from the surface of the cornea, to measure IOP. Upon initiation, the TonoLAB takes six IOP measurements in rapid succession (each a fraction of a second) by contacting the cornea and “rebounding”. Results are evaluated by internal software after removal of the high and low readings, with the average displayed. This process was repeated three times, and the average of those readings was recorded as the IOP of that eye. Isoflurane 0–5% was used to induce anesthesia for IOP measurements. Once the animal was induced, it was removed from the induction chamber and transferred to a nose cone with isoflurane 0–5% for maintenance, then placed on a heating pad with fleece blankets and monitoring equipment on a scissor lift stand. Refresh tears lubricant eye drops were placed in both eyes just prior to performing IOP measurements. IOP measurements may also be performed during imaging sessions.

### Ocular imaging

Tropicamide was given for dilation shortly after anesthesia induction. Tree shrews were placed on the imaging stage, which contains a warming plate, and moved near the imaging machine such that their eyes were nearly in contact with the imaging lens. GenTeal gel was applied to the eye to couple the eye with the lens. The imaging stand can be moved and adjusted to optimize the view of the fundus. Color fundus photography and OCT were completed first to remain free from dye-related artifacts. Then, for FA, fluorescein dye was injected intraperitoneally, with efforts to minimize movement of the shrew to maintain optimal imaging angles. The body suit was carefully unbuttoned while trying to leave the animal’s eye in view of the imaging scope. The right leg was lifted, and fluorescein was injected intraperitoneally. Video of the fundus was taken during fluorescein circulation for 6–10 minutes, or longer as needed to visualize ocular pathology.

### Radiation delivery

Radiation targeting was planned using bony landmarks as an initial localization step, with finalized targeting based on imaging the optic nerve and edge of the globe (Figure 4). Radiation targeting can be tailored to primarily study optic nerve injury, retinal injury, or more generalized globe injury, and appropriately targeted single fraction doses between 15 and 45 Gy are well-tolerated without dose-limiting toxicity. Radiation was delivered with a stereotactic animal irradiator using a 3.5 mm or 5 mm collimator and a 0 to 180 degree arc. Radiation was delivered under general anesthesia with a mixture of 2.5% vaporized isoflurane and pure oxygen, with animals immobilized using a stereotactic bite block. All irradiations were performed at 225 kVp, 20 mA, and 0.3 mm of Cu filtration using the X RAD SmART irradiator (Precision X-ray Inc., Madison, CT). Integrated cone beam computed tomography (CBCT) was used for targeting. Animals were placed in a prone position on the radiation stage. The irradiated left eye was aligned with lasers, using the lateral canthus as a landmark and moving the stage as needed to achieve optimal alignment of the eye in the planned treatment field. An initial CT scan was done for planning, followed by targeting with positional adjustments, re-targeting, and re-scanning as needed until optimal alignment. Final scans were saved to document animal positioning immediately prior to treatment delivery, and a final post-treatment CT scan was captured to confirm minimal movement during treatment.

### Intravitreal injection

**Figure 5:**
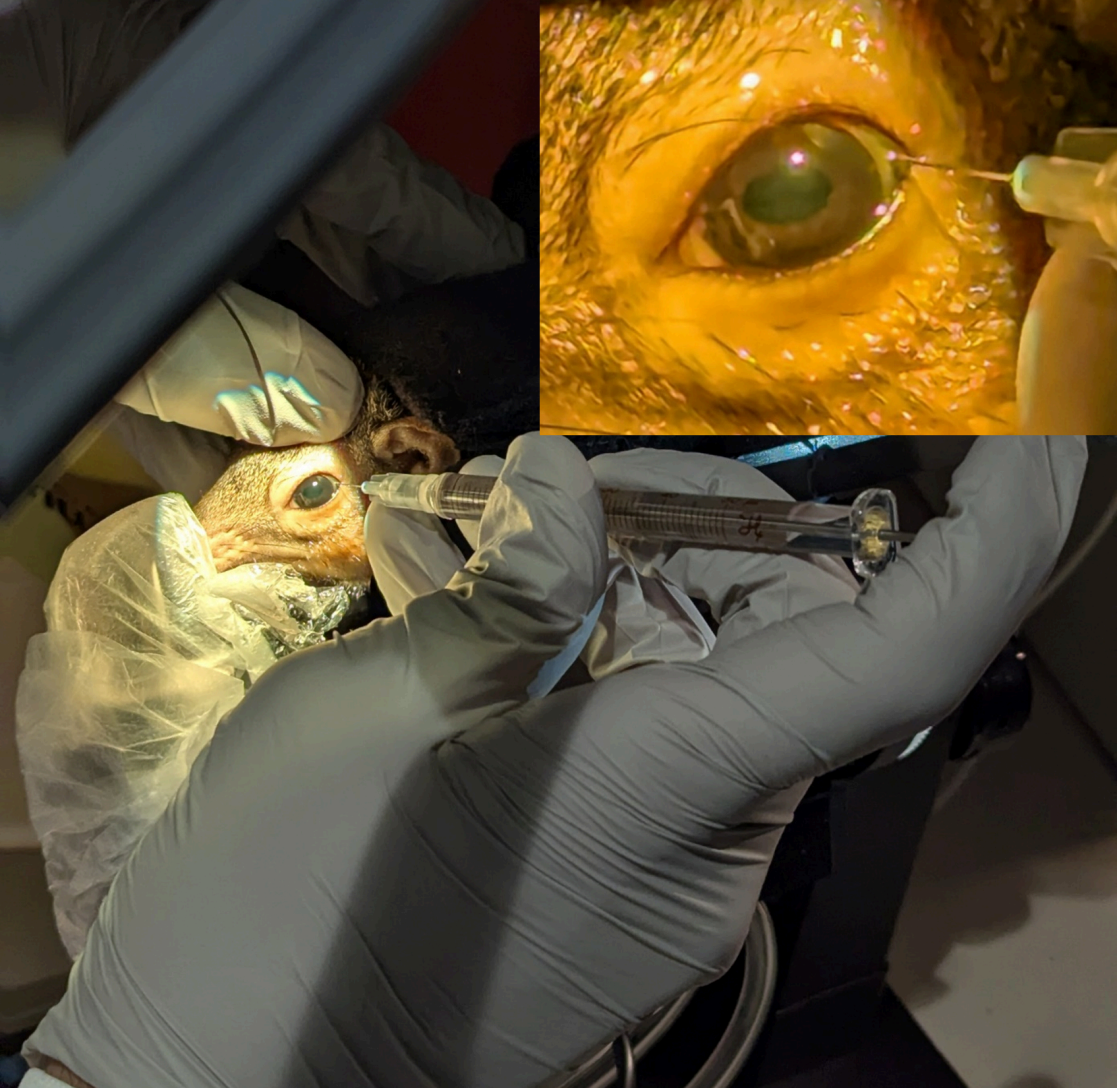
**Intravitreal injection into the tree shrew.** For intravitreal drug delivery, a needle was inserted approximately 2 mm posterior to the limbus and angled toward the optic nerve to enter the vitreous cavity and avoid puncture of the intraocular lens. The inset shows a magnified view of the injection location into the eye.

The shrew was positioned on its side with the treatment eye facing up. Injections can be done either with or without the aid of an operating microscope, but bright light is necessary to adequately see ocular structures in the dark tree shrew eye. The eyelids can be manually retracted, or a 0.12 forceps can be used to grasp the conjunctival tissue and rotate the eye to expose the temporal sclera. One drop of lidocaine was instilled for topical anesthesia, followed by one drop of 5% povidone iodine. A caliper was used to measure 2 mm posterior to the limbus. A 33 g needle on a Hamilton syringe was inserted in the superotemporal or inferotemporal quadrant and angled posteriorly toward the optic nerve (Figure 5). The drug can be injected with the help of an assistant to depress the plunger if necessary. We recommend a 5 µL volume for injection agents.

### Euthanasia

Following experimental endpoints, animals were euthanized by 5% isoflurane induction followed by 2 mL intraperitoneal euthanasia solution and secondary pneumothorax after death. If desired, blood can be drawn for laboratory analysis from the lateral saphenous vein following isoflurane induction, but prior to administration of euthanasia solution. After administration of the euthanasia solution, additional blood can be collected from the heart. Following secondary pneumothorax, the eyes and other desired organs or tissues can be collected for histopathology analysis.

## Expected results

### Tree shrew welfare can be enhanced through positive reinforcement target training

Positive reinforcement training using a finger or laser pointer improved tree shrew interactions with veterinary staff. While there was variability regarding handler approach latency, time to task completion, and presence of stereotypies, after 12 weeks of training, 11 of 12 (92%) animals were able to successfully target a laser pointer on the side of the cage compared to 7 of 12 on the first day of training (58%). Training appeared to increase animal approach to handlers, with all animals (100%) approaching and accepting treats by hand at 12 weeks compared to 5 of 12 on the first day of training (42%). This suggested a behavioral shift, demonstrating increased trust in handlers, which is beneficial for animal welfare.

### IOP in the tree shrew is more reliably measured using inhalational anesthesia alone

**Figure 6:**
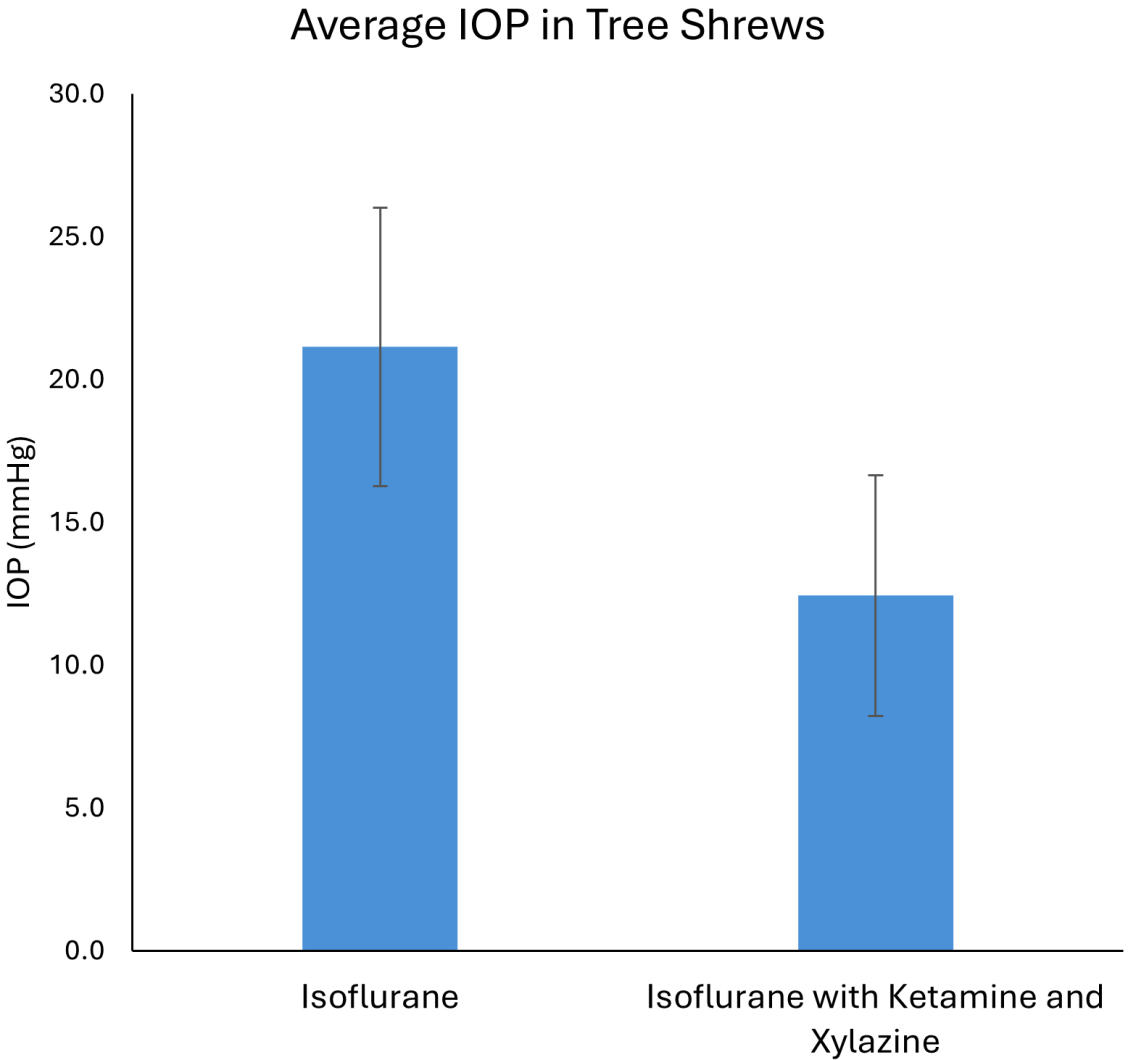
**Intraocular pressure measurement in the tree shrew.** Intraocular pressure measurements were closer to the expected normal range (21.2 ± 4.9 mmHg) using inhalational anesthesia alone (isoflurane). Using a combination of inhalational and injection anesthesia (isoflurane with ketamine and xylazine), intraocular pressure measurements appeared artificially low (12.4 ± 4.2 mmHg, *p* < 0.001). Data were collected on *n* = 6 animals, each with bilateral eye measurements, with each animal serving as its own paired control for the two distinct anesthetic conditions.

We measured IOP using both an isoflurane-only method and an isoflurane plus injectable anesthesia method (used during imaging). We noted that IOP readings were closer to an expected normal range under isoflurane (inhaled) anesthesia alone, while IOP seemed artificially low when using injectable plus isoflurane anesthesia (Figure 6). Therefore, we recommend inhalational anesthesia alone if performing studies that require accurate IOP assessment.

### A tree shrew model of ORI is feasible and demonstrates hallmarks of human disease

**Figure 7:**
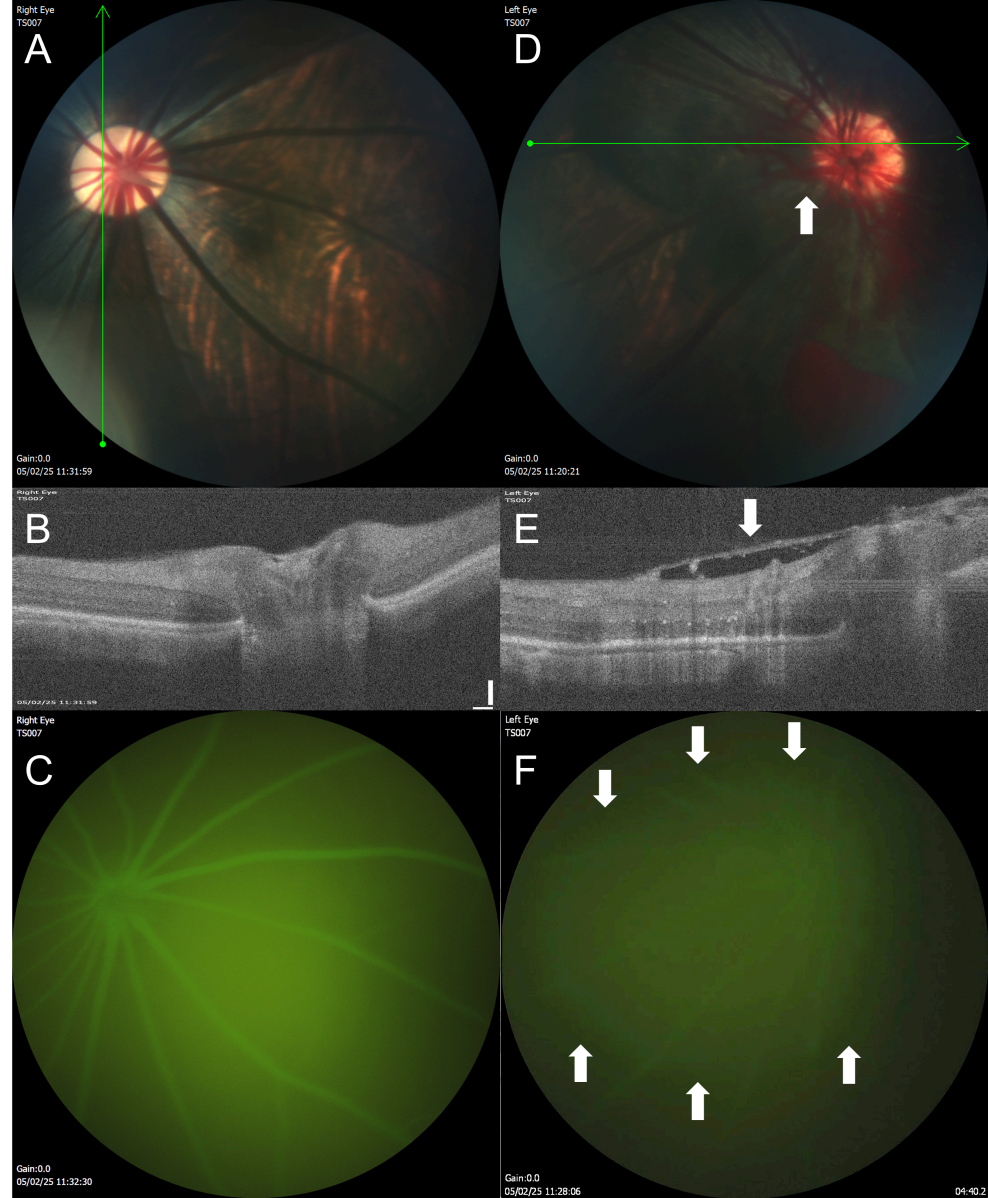
**Ocular imaging in the tree shrew model of ocular radiation injury.** (**A**) The untreated right eye of the tree shrew (TS007) has healthy-appearing retinal vasculature and optic disc. (**B**) On optical coherence tomography (OCT) taken through the area of the green line shown on the fundus photograph in A, the retinal layers can be clearly visualized in different shades of white and gray, with the optic nerve depression near the center of the image. (**C**) The retinal vasculature shows normal filling with no leakage by fluorescein angiography (FA). (**D**) The irradiated left eye of the same animal, 9 months after treatment, has developed neovascularization (arrow) with retinal hemorrhages seen on fundus photography. (**E**) By OCT, a fibrovascular membrane (arrow) has formed overlying the retina, and the normal optic nerve head depression is absent. (**F**) By FA, the view is hazy due to leakage (area surrounded by arrows) from the neovascular vessels.

Following radiation targeting to the posterior segment of the eye, tree shrews developed hallmarks of ORI within 6–9 months (Figure 7). Expected findings include the development of cotton wool spots (fluffy, white areas that indicate swelling in the retinal nerve fiber layer) and retinal hemorrhages. With more advanced ORI, neovascularization can be seen. We recommend grading severity of ORI using a modified clinical grading system from 0–4 defined as (0) no intraocular radiation side effects, (1) hemorrhages or cotton wool spots in one-two quadrants of the retina, (2) hemorrhages or cotton wool spots in three-four quadrants, (3) retinal nonperfusion, or (4) retinal neovascularization [16].

### Conclusion

The tree shrew is a promising model for human disease research, including ophthalmic applications. Optimization of animal handling and interventions is necessary to enhance animal welfare and procedural intervention. The tree shrew can be used to generate a model of ORI with clinical features that may also be seen in human patients. Such models will permit testing of novel therapeutic strategies, providing preclinical data for treatments that may better preserve vision in patients undergoing radiotherapy and supporting translation to successful clinical trials. In summary, this model will aid in future translational research efforts to uncover novel strategies for the prevention or treatment of ORI, which will improve vision-related quality of life for patients who require radiation treatment for cancers in the head and neck region.

## Abbreviations

FA: fluorescein angiography
IOP: intraocular pressure
OCT: optical coherence tomography
ORI: ocular radiation injury

## Declarations

### Availability of data and materials

The datasets that support the findings of this study are available from the corresponding author upon reasonable request.

### Copyright

© The Author(s) 2025.
